# Improved Hydrological Decision Support System for the Lower Mekong River Basin Using Satellite-Based Earth Observations

**DOI:** 10.3390/rs10060885

**Published:** 2018-06-06

**Authors:** Ibrahim Nourein Mohammed, John D. Bolten, Raghavan Srinivasan, Venkat Lakshmi

**Affiliations:** 1 Science Applications International Corporation, Hydrological Sciences Laboratory, NASA Goddard Space Flight Center, Mail Code 617.0, Greenbelt, MD 20771, USA; 2 Hydrological Sciences Laboratory, NASA Goddard Space Flight Center, Mail Code 617.0, Greenbelt, MD 20771, USA; 3 Spatial Sciences Laboratory, Department of Ecosystem Science and Management, Texas A&M University, College Station, TX 77843, USA; 4 School of Earth Ocean and Environment, University of South Carolina, Columbia, SC 29208, USA

**Keywords:** Mekong River, water balance, remote sensing, SWAT, streamflow

## Abstract

Multiple satellite-based earth observations and traditional station data along with the Soil & Water Assessment Tool (SWAT) hydrologic model were employed to enhance the Lower Mekong River Basin region’s hydrological decision support system. A nearest neighbor approximation methodology was introduced to fill the Integrated Multi-satellite Retrieval for the Global Precipitation Measurement mission (IMERG) grid points from 2001 to 2014, together with the Tropical Rainfall Measurement Mission (TRMM) data points for continuous precipitation forcing for our hydrological decision support system. A software tool to access and format satellite-based earth observation systems of precipitation and minimum and maximum air temperatures was developed and is presented. Our results suggest that the model-simulated streamflow utilizing TRMM and IMERG forcing data was able to capture the variability of the observed streamflow patterns in the Lower Mekong better than model-simulated streamflow with in-situ precipitation station data. We also present satellite-based and in-situ precipitation adjustment maps that can serve to correct precipitation data for the Lower Mekong region for use in other applications. The inconsistency, scarcity, poor spatial representation, difficult access and incompleteness of the available in-situ precipitation data for the Mekong region make it imperative to adopt satellite-based earth observations to pursue hydrologic modeling.

## 1. Introduction

The complexity of managing water resource, e.g., the Mekong River, stems from the fact that there are many competing interests, such as societal, cultural, economic, and environmental interests, that all need to be synchronized to achieve the goal of prosperity and sustainability [1–6]. Sivapalan and Blöschl [7] argued that the role of finer resolution remote sensing data and models that represent catchments as complex systems and that link time scales, is the notion that is most common in the current era (2010–2030) to address contemporary hydrological challenges. Growing populations, sustaining socio-economic activities (e.g., fishery), ecological needs, the effects of climate change, and energy security are some of the complex challenges experienced in the Mekong River Basin [8–13]. As a consequence, utilizing newly-developed remote sensing products and modeling to address the Mekong River is vital.

This work integrates multiple satellite-based earth observation systems, in-situ station data and spatial data with the Soil & Water Assessment Tool (SWAT) hydrologic model employed in the Mekong River Basin region to improve the Lower Mekong River Basin region’s hydrological decision support system, based on both hydrological flow and total water demand/use. The scarcity and the incompleteness of the data observations from many stations make it imperative to use satellite-based remote sensing data when modeling the hydrological fluxes in the Lower Mekong River Basin (LMRB). This work has developed a comprehensive suite of hydrological data products that can be used to improve water accounting and floodplain management using hydrological cycle variables such as runoff, evapotranspiration, and precipitation in the LMRB. The main objective of this work is the improvement of the hydrological decision support system for the Lower Mekong River Basin. This work explores streamflow simulation for the Lower Mekong River by examining the usability of satellite-based remote sensing data products, comparing them to the traditional in-situ station data. Overall, our work aims to assess the value-added information from the simulation of hydrological processes in the LMRB by using SWAT with climatological forcing data from satellite-based earth observations as an alternative to scarce in-situ station data.

## 2. Materials and Methods

### 2.1. Study Area

**Figure 1:**
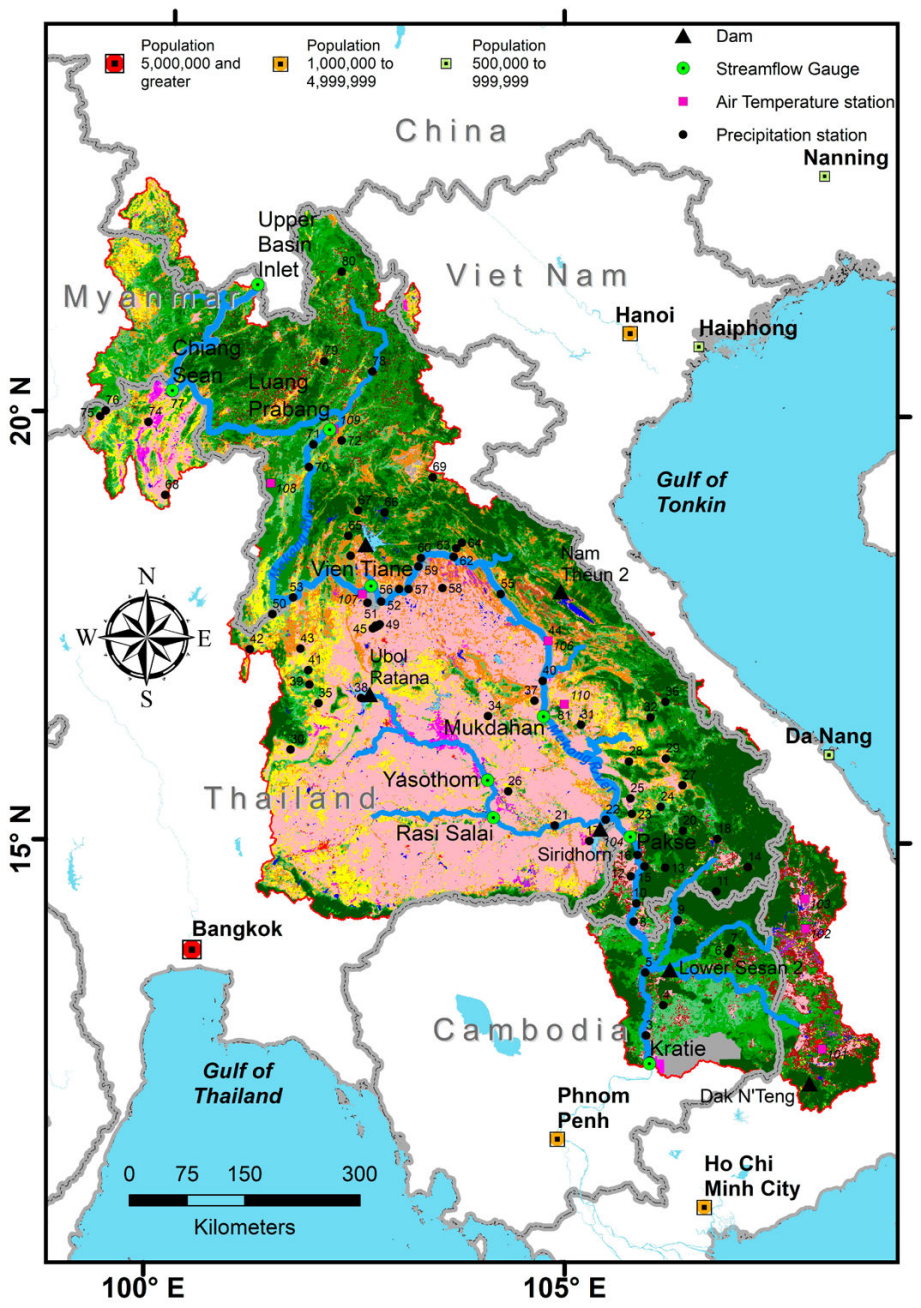
The Lower Mekong River Basin. Streamflow gauges (described in Table 1) follow the Lower Mekong River Basin subareas, as presented by Rossi et al. [14]. Cities with population classes obtained from the Environmental Systems Research Institute, Inc. (ESRI) World Populated Places layer (https://www.arcgis.com/home, accessed on 25 May 2018) are depicted in red (greater than 5 million), orange (1–5 million), and light green (0.5–1 million).

The Mekong River originates in the high altitudes of the Tibetan Plateau in China and flows south through five countries (Myanmar, Lao People’s Democratic Republic (PDR), Thailand, Cambodia, and Vietnam) ending in a large delta before exiting to the South China Sea. The Mekong River Basin is divided into upper and lower basins. The Lower Mekong River Basin begins when the Mekong River leaves the Chinese province of Yunnan and enters the Golden Triangle, where the borders of Thailand, Lao PDR, China and Myanmar come together (Figure 1).

### 2.2. Spatial Data

A digital elevation model (DEM) with 1” (one arcsec) grid resolution for the study area was obtained from the Advanced Spaceborne Thermal Emission and Reflection Radiometer (ASTER) Global Digital Elevation Model (https://doi.org/10.5067/ASTER/ASTGTM.002). The DEM map with 90-m resolution was used to derive the slope and aspect grids for the LMRB model input. The slope class of 2–8% covers about 40% of the watershed area. The LMRB topography ranges from 2838 m above sea level in the Annamese Cordillera mountains range, Laos (the Mekong River and the South China Sea are boundaries) to 89 m below sea level (outlet) with a mean elevation over the basin of 479 m.

The study area soil information data was obtained from the Harmonized World Soil Database (HWSD) [15]. The LMRB soil texture is mainly sandy clay loam and covers approximately 42% of the basin.

The land use/land cover (LULC) data was obtained from a 2010 LULC map at a spatial resolution of a 0.25 km for the Lower Mekong Basin using 2010 Moderate Resolution Imaging Spectroradiometer (MODIS) monthly Normalized Difference Vegetation Index (NDVI) data as the primary data source [16].

The study watershed LULC areas are mainly forest and agricultural lands. Rice is farmed on about 26% of the watershed area, while forest land cover constitutes about 30% of the watershed area [16].

### 2.3. In-Situ Data

The discharge data for this work was obtained from the Mekong River Commission (MRC, www.mrcmekong.org). Updated discharge data was interpolated from recent observed level data obtained from the Asian Preparedness Disaster Center (ADPC, www.adpc.net). Data for existing dams within the LMRB were obtained from the Greater Mekong Consultative Group for International Agricultural Research (CGIAR) Research Program on Water, Land and Ecosystems [17]. In Figure 1, we depict dams within the LMRB that are either already commissioned or still under construction and have a maximum reservoir area greater than or equal to 280 km^2^, similar to the MRC Mekong River model setup. The surface area of the reservoirs behind the various dams that we included in this study as well as the geographic annotations for the in-situ stations depicted in Figure 1, are visualized interactively with the compressed keyhole markup language files (KMZ) Supplementary Materials of this manuscript.

**Figure 2:**
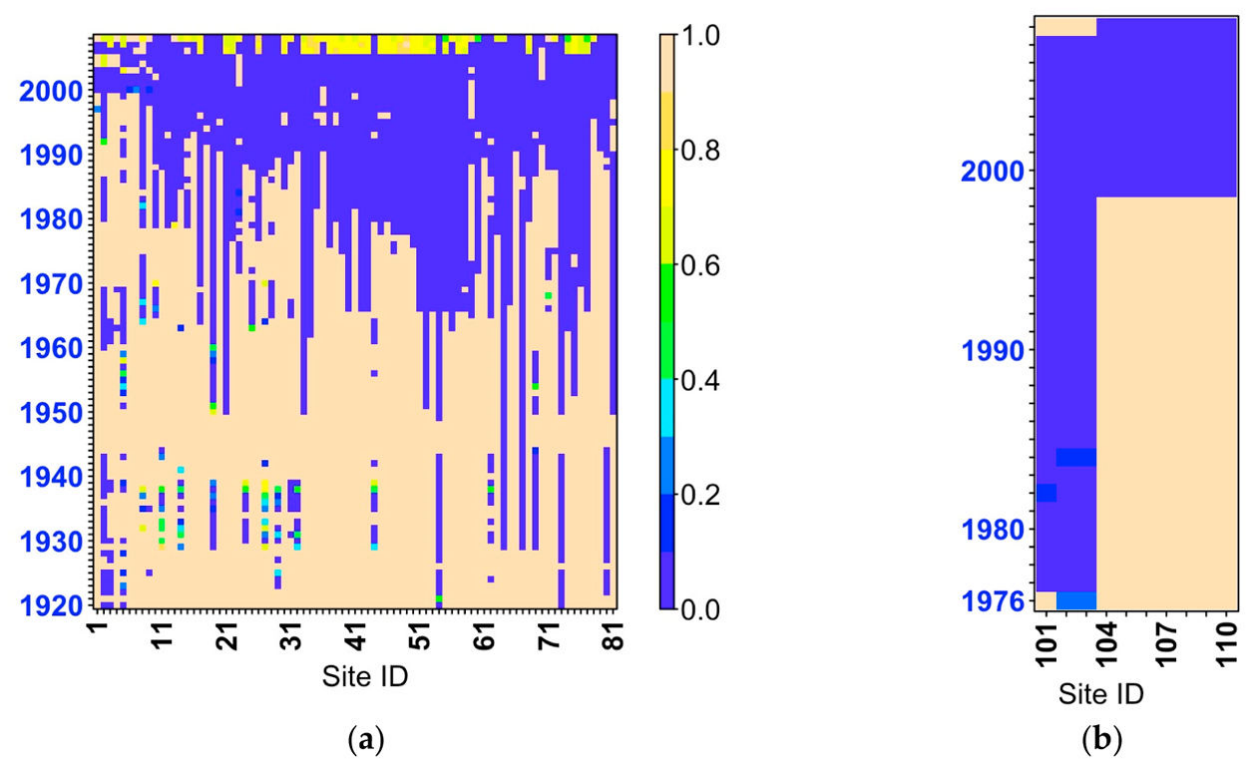
(**a**) Precipitation (81 stations), and (**b**) air temperature (10 stations) in situ data availability at the Lower Mekong Basin. The dark blue color (value of 0 or 0%) refers to a complete data record, while the beige color (value of 1 or 100%) refers to a complete missing data records during a specific year for a specific site.

Daily precipitation and minimum and maximum air temperature data was obtained from the Mekong River Commission data respiratory. Figure 2 depicts the in-situ data availability in the Lower Mekong Basin. We note that precipitation data are available for some sites since 1920 and started to cover the whole basin around 1990s. Air temperature data (minimum and maximum) are available at fewer sites than precipitation.

### 2.4. Meteorological Data

**Figure 3:**
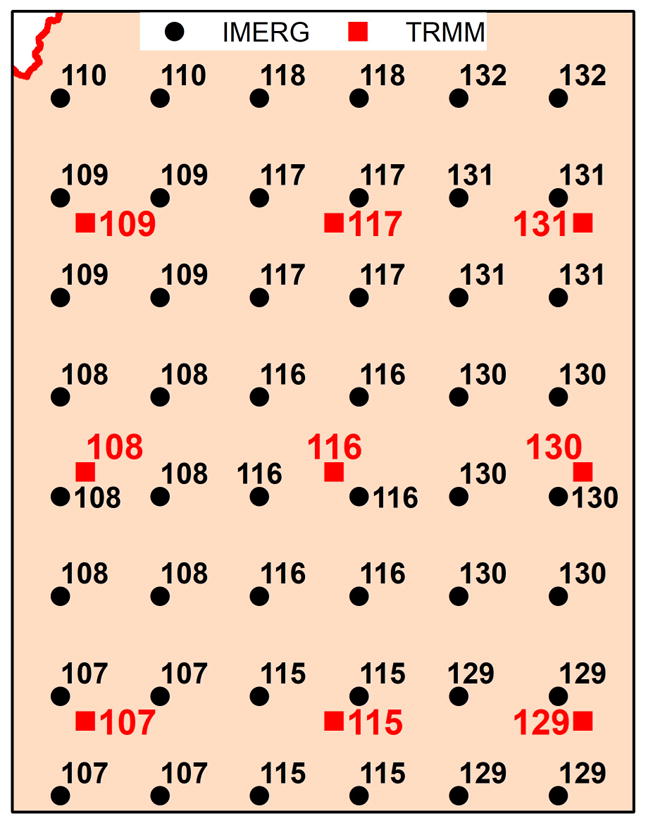
A schematic showing a layout of the Tropical Rainfall Measurement Mission (TRMM) (rectangle in red color at a spatial resolution of 0.25 deg.) and the Integrated Multi-satellite Retrievals for the Global Precipitation Measurement mission (IMERG) (circular in black color at a spatial resolution of 0.1 deg.) grids labelled with numbers illustrating nearest neighbor connectivity. TRMM data from March 2000 to March 2014 were used to fill the IMERG grids following the joining methodology explained.

Daily cumulative precipitation data was obtained from the Global Precipitation Measurement mission (GPM) and the Tropical Rainfall Measurement Mission (TRMM) remote sensing data and used as inputs for the LMRB model. The Integrated Multi-satellite Retrieval for the Global Precipitation Measurement mission (IMERG) dataset used for this work was the *GPM_3IMERGDF* (https://pmm.nasa.gov/data-access/downloads/gpm). Since IMERG data products are only available from 12 March 2014 to present, we used the TRMM rainfall data (*3B42RT*) for time periods earlier than 12 March 2014. A nearest neighbor methodology was used to fill the IMERG data points with the TRMM data points as an approximation during the 1 March 2000 to 11 March 2014 time period, because the TRMM and IMERG data do not have the same spatial resolution (i.e., 0.25 and 0.1 degree respectively). The Euclidean or great circle distance was calculated between the TRMM and IMERG cell centroids to achieve filling data points. The minimum distance between the IMERG and TRMM points was used for the filling assignment. A layout showing part of the IMERG and TRMM cell points within the LMRB, labelled with identification numbers that reflect the assignment, is illustrated in Figure 3.

Minimum and maximum daily air temperature data was calculated from the air temperature records obtained from the Global Land Data Assimilation System (GLDAS) simulation data products [18]. For this work, we used the *GLDAS_NOAH025_3H.2.1* data products retrieved from https://disc.gsfc.nasa.gov/. The wind speed, relative humidity, and solar radiation data needed for our modeling work was estimated using the global reanalysis weather data from the National Centers for Environmental Prediction (NCEP, http://www.ncep.noaa.gov/), and the Climate Forecast System Reanalysis (CFSR).

### 2.5. Hydrological Model—SWAT

The SWAT is a conceptual watershed-scale hydrological model designed to address challenges related to water management, sediment, climate change, land use change, and agricultural chemical yield [19–24]. The SWAT applications range from the field scale to the watershed scale [25] to the continental scale [26,27]. The SWAT model components are hydrology, weather, sedimentation, soil temperature, crop growth, nutrients, pesticides, and agricultural management. The hierarchical structure for modeling units in SWAT is set to be multiple sub-watersheds, which are then further subdivided into hydrological response units (HRUs) that consist of homogeneous land use, management, and soil characteristics. The SWAT simulates the overall hydrological balance for each HRU and model output is available in daily, monthly, and annual time steps. SWAT meteorological inputs include daily precipitation, maximum and minimum temperature, solar radiation, humidity and wind speed. The version of SWAT used in this work was SWAT2012 rev. 635 [28]. The Penman–Monteith method was used to simulate potential evapotranspiration for this work. The SWAT Calibration and Uncertainty Procedures (SWAT-CUP) software package with the Sequential Uncertainty Fitting (SUFI2) method [29] was used for model calibration. The watershed stream network and sub-basins were generated using the Arc SWAT software (http://swat.tamu.edu/software/arcswat/) watershed analysis module (watershed delineator) with a contributing area threshold of 253.5 km^2^, resulting in 1138 sub-basins. Applying the HRU module in the Arc SWAT software with 10% land use percentage over the sub-basin area, 10% soil class percentage over the land use area, and 10% slope class percentage over the soil area, we obtained 10,096 HRUs for the LMRB model.

## 3. Results and Discussion

This study highlights the benefit of satellite-based earth observations data for hydrological modeling in regions that experience poor spatial in-situ earth observations data representation. It has been well established in literature that climate forcing data is the dominant contributor in determining the hydrologic response. The ability of our developed hydrological model to represent the variability of the observed discharge at multiple sites along the Lower Mekong River when driven by satellite-based earth observation data corroborates the role of quality climate forcing as one of the main determinants in hydrologic modeling.

We assessed the performance of satellite-based data products (TRMM and GPM) in estimating precipitation and streamflow over the Lower Mekong River Basin. We noticed that the quality of the satellite-based remote sensing precipitation data, especially in the southern part of the LMRB (i.e., close to the delta) was better than elsewhere in the basin. This finding could be used for the further refinement of satellite-based remote sensing products in the Lower Mekong region. It is worth mentioning here that although our calibration and validation work (Cal/Val) was done for years before the onset of GPM data, the simulated discharge results driven by the GPM precipitation data using the precipitation adjustment parameters obtained by the Cal/Val work were promising and matched the observed discharge values along the Lower Mekong River.

### 3.1. LMRB Water Balance

**Figure 4:**
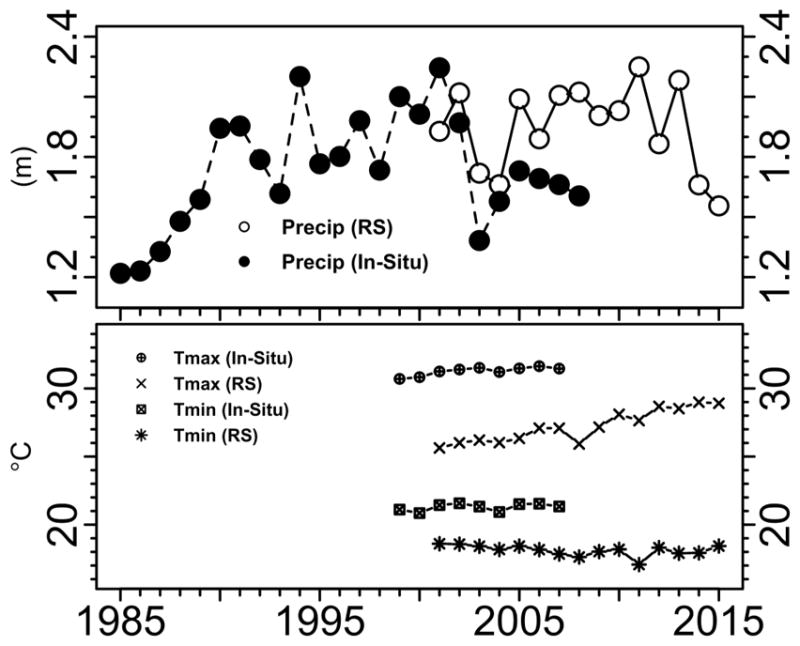
The Lower Mekong River Basin time series data. Annual aggregated weighted average precipitation in meters over the LMRB using satellite-based remote sensing (RS) and in-situ data. Mean annual aggregated weighted air temperature pattern (maximum and minimum air temperatures) in degrees Celsius using remote-sensing-calculated products and in-situ data.

The average annual precipitation in the study watershed during 2001–2015 was 1.9 m (satellite-based remote sensing data products). The average maximum annual air temperature in the study watershed during 2001–2015 was 27 °C, while the average minimum annual air temperature during 2001–2015 was 18 °C (GLDAS data products). We also note here there is about 2 °C difference between air temperature estimates using GLDAS data products and in-situ station data products. We believe that in-situ air temperature station data does not represent the entire watershed accurately, since there is a bias attributed to location and availability. We summarize the precipitation and air temperature annual information for the study watershed from 1985–2015 in Figure 4.

Table 1 gives various statistical measures for the Lower Mekong River annual discharge using calendar years at different gauges along the main stem river and upstream tributaries. The upper basin inlet discharge record for the years 2008 and onward, required for our modeling work, were regressed from the nearby station (Chiang Sean) discharge record. The Vientiane (Lao PDR) station # 011901, which has the longest available monitoring record compared to the other stations studied (1913–2016), has a mean annual discharge of 4476 m^3^/s. Minimum, maximum, different quantiles, standard deviation, and coefficient of variation values for annual discharge at different stations are presented. Discharge station skewness values suggest the location and the shape of the probability distribution (i.e., positive or negative). In Table 1, we also provide the Hurst coefficient [30,31]. The Hurst coefficient is an indicator of a serial correlation or dependence for the annual discharge time series studied. Across the multiple discharge stations studied in the Lower Mekong, the Hurst coefficient for annual flow was greater than 0.5, suggesting that high flow will most likely be followed by another high flow in the future. Multiple works have presented various LMRB discharge statistics [14,32]. However, Table 1 adds new information—the coefficient of variation, skewness, and persistence and autocorrelation explained by the Hurst coefficient for the Lower Mekong River. Discharge statistics at nine streamflow gauges representing the outlets of eight sub-basins in the Lower Mekong River Basin, in addition to the Upper Mekong River inlet, are provided in Table 1. The geographic locations of these nine streamflow gauges are referenced in Figure 1 with a green filled circle symbol.

### 3.2. Calibration and Verification of the LMRB Model Using TRMM

SWAT uses many parameters to describe typical soil, plant growth, land cover, reservoir, and agricultural management characteristics. In this work, the LMRB model was calibrated to the monthly average discharge at the LMRB sub-basin outlets during the 2005 and 2006, with a few parameters as outlined in Table 2. The reason for selecting 2005 and 2006 as the calibration years was based on the fact that the precipitation amounts in these two years were close to the average annual precipitation over the Lower Mekong River basin. The validation of the LMRB model was performed at the LMRB sub-basin outlets during the time period 2001–2004, and in 2007. The validation time period was picked so that a common time period for the available satellite-based remote sensing and in-situ climate forcing data exists. The availability of remote-sensing, satellite-based and in-situ air temperature and precipitation forcing data is depicted in Figure 4. The parameters used and suggested range values for the LMRB model calibration were consulted and obtained from SWAT developers (*R. Srinivasan, personal communication*) and the previous works of Neitsch et al. [33] and Rossi et al. [14]. All other parameters in the LMRB model were left at their default values. In Table 2, we provide parameter-calibrated values for the two models that we performed (model forced with satellite-based remote sensing precipitation and in-situ precipitation). Three groups of parameters related to precipitation, high flow, and base flow are presented in Table 2. The range of the correction factor to grid precipitation shown in Table 2 are the values used in SWAT-CUP to adjust the forcing precipitation data (i.e., *increment*). The calibrated values for the soil evaporation compensation factor parameter (ESCO = 0.6 and 0.75) were found to be lower than the previous values reported by Rossi et al. [14] for the LMRB. Generally, as the value for ESCO is reduced, the SWAT model is able to extract more of the evaporative demand from lower soil layers. We argue here that the newer soil data used in this work has influenced a newer ESCO value for the LMRB that is different from the default value previously used (i.e., ESCO = 0.95). The parameters listed in Table 2 are among many parameters that describe the SWAT soil physical characteristics and influence the movement of water and air through the soil profile and shallow aquifer underneath it, thus they have a major impact on the cycling of water within the SWAT modeling unit (HRU).

**Figure 5:**
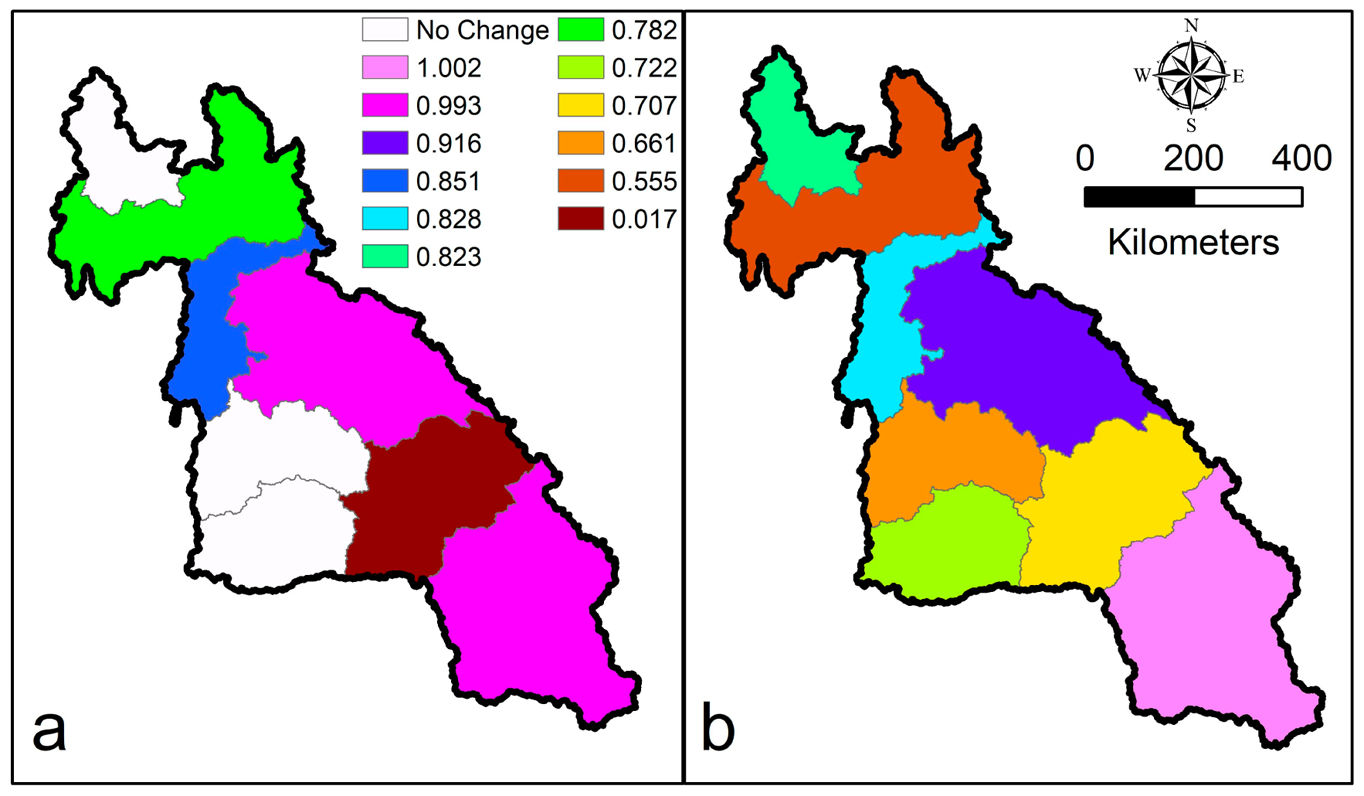
Precipitation data adjustment layout for (**a**) in-situ station, and (**b**) satellite-based remote sensing (RS) model input. The precipitation data adjustment equals to 1+*increment*, as outlined in Table 2.

Our hydrological model showed higher sensitivity to parameters related to correction adjustment factors for precipitation forcing inputs. Figure 5 provides the LMRB model precipitation forcing adjustment factor layout for the in-situ and remote-sensing, satellite-based precipitation datasets. This layout could be used to guide the correction of TRMM and GPM earth observations for future applications in the region. Polygons with no change in precipitation forcing adjustment (i.e., shown in white color) indicate the non-existence of rain stations or rain station exclusion due to the incompleteness of data records (Figure 5a). This matches earlier observation seen in Figure 1 that there are a few number of rain stations over the LMRB. We note here that a sequential calibration procedure was performed starting from sub-basin outlet 1 and going downstream till sub-basin outlet 6 (sub-basins 7 and 8 are the western tributary outlets for the Mekong River and are draining to sub-basin 5). This has resulted in the production of the correction adjustment factor layout in polygons corresponding to sub-basin outlets. Figure 5 serves as a quality check for the precipitation forcing data in the LMRB when applied in hydrological applications. Precipitation forcing data from satellite-based remote sensing tend to be more skewed in the northern part of the LMRB in comparison with the southern part. In general, we found that running our model without satellite-based remote sensing precipitation data adjustments tended to overestimate the simulated discharge by about 13%.

**Figure 6:**
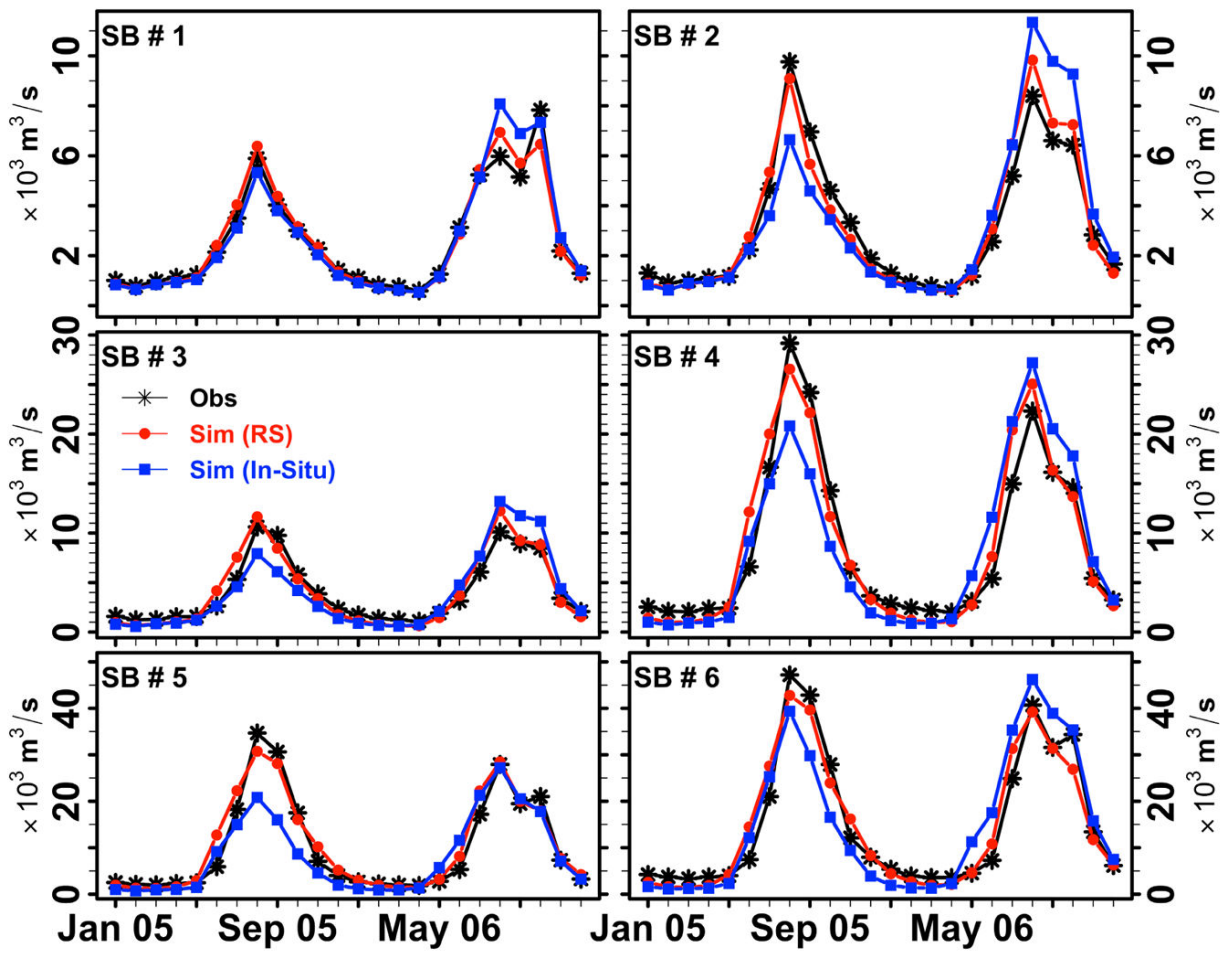
The LMRB model calibration. Monthly mean observed and simulated discharge in m^3^/s at six sub-basin watersheds in calibration of the LMRB model. The calibration time period is from 2005 to 2006. The red circles are simulated discharge with satellite-based, remote-sensing precipitation data input, while the blue rectangles are simulated discharge with in-situ meteorological data input. Here SB stands for sub-basin.

Figure 6 depicts the LMRB model performance during the calibration years (2005, 2006), driven by satellite-based remote sensing earth observations (blue rectangle) and in-situ (red circle) meteorological data at six watershed outlets within the Lower Mekong Basin. The simulated discharge results obtained from satellite-based remote sensing data were able to explain more than 91% of the variance observed in the monthly discharge during the calibration years (i.e., the Nash–Sutcliffe Efficiency (NSE) varied from 0.91 to 0.96 from sub-basin 1 to 6). The LMRB model performance when driven with in-situ data was able to explain from 68% to 91% of the variance observed in the monthly discharge during the calibration years. The LMRB model overestimated the monthly discharge by about 5% at sub-basin 5 and underestimated the monthly discharge by about 2% at sub-basin 6 during the calibration years when driven by satellite-based remote sensing data. Table 3 provides the calibration metrics used to assess the performance of the LMRB model when driven by satellite-based remote sensing and n-situ data. The percent error (Q_err_) between the monthly mean simulated and observed discharge and the NSE performance metrics are tabulated for the LMRB model under satellite-based remote sensing and in-situ data (Table 3). We note here that the results shown in Figure 6 and Table 3 suggest that the simulated model discharges utilizing satellite-based remote sensing data inputs are able to capture the variability of the observed streamflow patterns in the Lower Mekong better than simulated model discharges forced with in-situ data. We also note that the Q_err_ at SB5 and SB6 (LMRB outlet) were higher than other outlets examined in the LMRB calibration work (Table 3). We think that the differences in Q_err_ among the sub-basins can be attributed to the sequential calibration method that we used in this work, and the interaction of the dam release rules observed in the basin. In summary, our LMRB model evaluation results are similar to previous attempts presented by Rossi et al. [14], who reported Nash–Sutcliffe flow monthly efficiency values ranging between 0.8 and 1.0 at mainstream monitoring stations.

**Figure 7:**
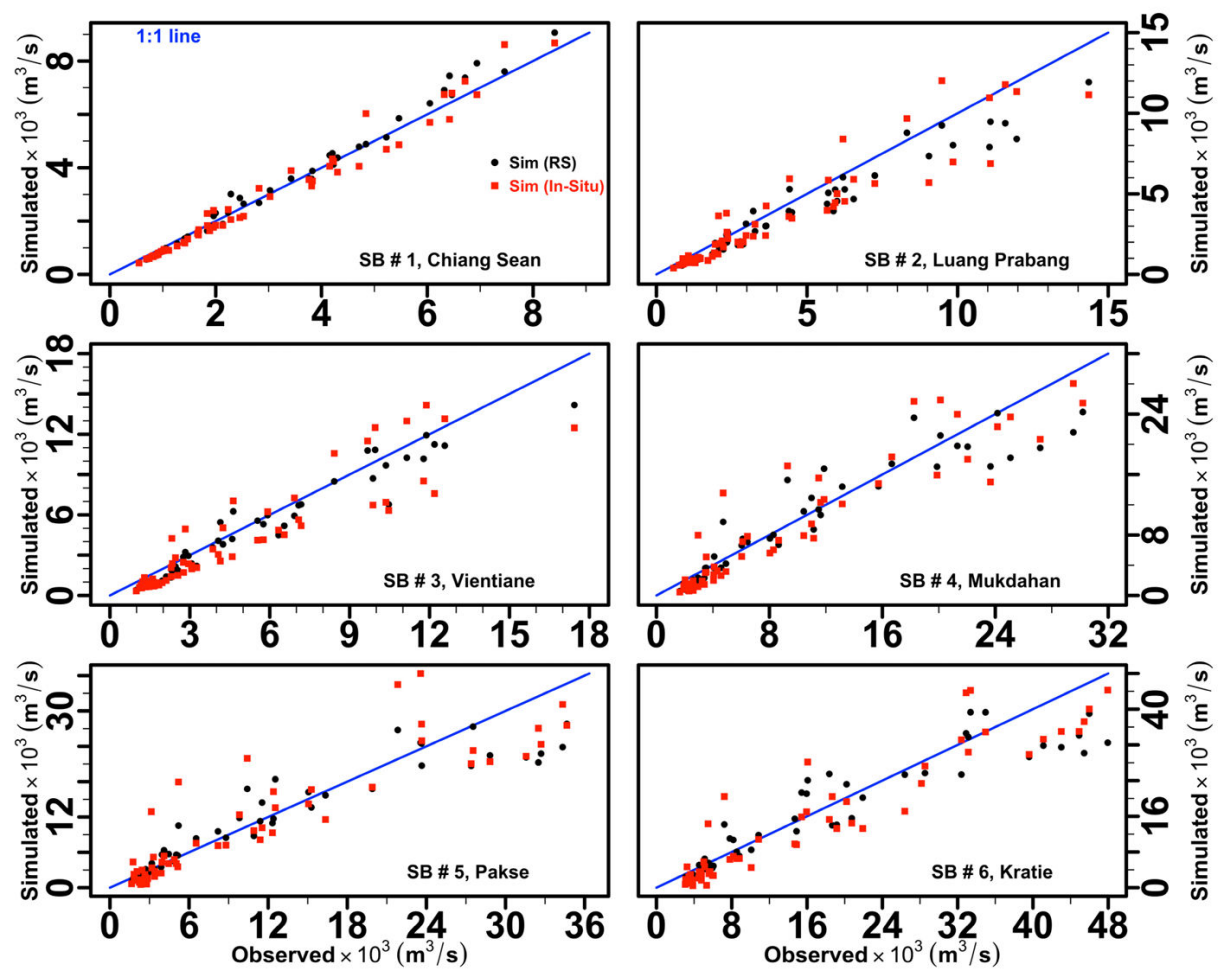
The LMRB model verification. Scatterplot of monthly observed and simulated discharge in m^3^/s for the Lower Mekong River at six sub-basin watersheds in validation of the LMRB model for 2001–2004, and 2007. Black circles indicate simulated discharge with satellite-based remote sensing meteorological data input, while red rectangles indicate simulated discharge with in-situ precipitation and air temperature data input.

Figure 7 provides monthly observed and simulated discharge for the study watershed for the validation of the LMRB model over five years (2001–2004, and 2007). Black circles and red rectangles were used to distinguish between the simulated discharges generated using satellite-based remote sensing and in-situ data as forcing inputs. In general, the model captured the timing of the onset and end of seasonal discharge but was slightly off in some estimates of peak flow. The NSE metrics during the validation time period for our model driven by satellite-based remote sensing climate data (RS) and in-situ climate data (In-Situ) are provided in Table 4. We note that the NSE performance metrics for our model varied between 0.88 and 0.98 when driven by satellite-based remote sensing climate data and between 0.75 to 0.97 when driven by in-situ climate data. The model had about 3.85% error on average in estimating monthly flows during the validation time period.

### 3.3. Verification of the LMRB Model Using GPM

**Figure 8:**
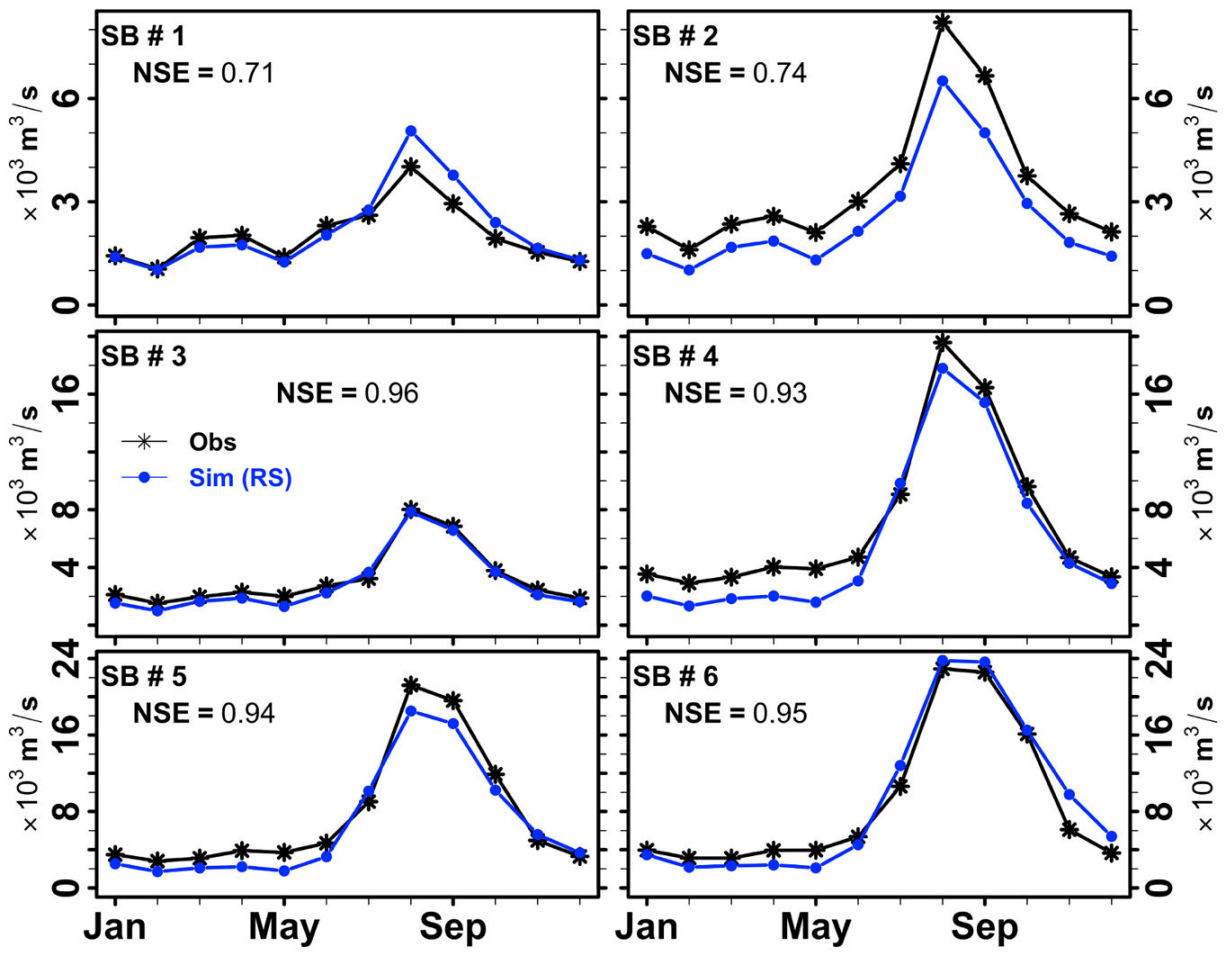
Monthly mean observed and simulated discharge in m^3^/s at six sub-basin watersheds with the use of the LMRB model for 2015. The IMERG precipitation data used to drive the LMRB model. The Nash–Sutcliffe Efficiency (NSE) performance metrics are depicted for each sub-basin. Here SB stands for sub-basin.

Figure 8 depicts the ability of the LMRB model to simulate discharge at various sub-basin outlets using GPM-IMERG precipitation as the forcing climate data. The simulated discharge results in Figure 8 were able to explain between 71% to 96% of the variance observed in monthly discharge during the year 2015 (sub-basin 1 to sub-basin 6). We note here that there is a slight difference in the LMRB model performance results when we compare the LMRB model forced with GPM-IMERG and TRMM-3B42RT (Figures 6 and 8). We attribute the model performance difference to the adjustments that we made to the precipitation forcing data to calibrate the LMRB model. We think that the adjustments we made to the TRMM-3B42RT forcing data during calibration years and verified in the verification years could be also used reasonably by the LMRB model when forced with GPM-IMERG data.

### 3.4. Nasaaccess Tool

We developed a ‘nasaaccess’ package (version 1.2) within the R software framework [34] to streamline the accessing and processing of the National Aeronautics and Space Administration (NASA) earth observation data products (i.e., TRMM, GPM, and GLDAS). Our package incorporates the methods we introduced (Figure 3) to address the spatial scale issues seen between TRMM and GPM. The ‘nasaaccess’ package creates weather input definition tables as well as station data files in a format readable by the SWAT model or any other rainfall/runoff model. The ‘nasaaccess’ package can be expanded to include other earth observation data products needed in the future. For the time being, ‘nasaaccess’ generates the daily rainfall and minimum and maximum air temperature gridded data and gridded data definition files needed to serve as a setup to run any basic SWAT/other model. The core functionality of the ‘nasaaccess’ package can be summarized by the following steps:

- Access the NASA Goddard Space Flight Center (GSFC) servers to download earth observation data,
- Clip needed grids based on a user study watershed input shapefile,
- Handle temporal and spatial issues (e.g., the GLDAS product has 3-h temporal resolution),
- Generate daily climate gridded data files and definition files compatible with SWAT/other models.

In summary, the inputs needed for the various functions within the ‘nasaaccess’ package are: start and end dates for the user’s required earth observation data, a shapefile for the study area of interest, and a DEM grid for the area of interest. The ‘nasaaccess’ package was used effectively in this work to process the meteorological input data for the LMRB model.

## 4. Conclusions and Recommendations

In this work, we showed that earth observation data enabled us to develop a regional hydrological decision support system application for the Lower Mekong River Basin. The inconsistency, scarcity, poor spatial representation, as well as difficult access and incompleteness of the available in situ data in the Mekong region make it absolutely imperative to adopt earth observation data products to pursue hydrological modeling for the Lower Mekong River Basin. We also introduced a smoothing technique method to address the spatial scale issues observed in the TRMM and GPM earth observation data. We also produced a software tool that can be used to access and process earth observation data products on a global scale.

The use of satellite and field data has helped us to evaluate and improve a hydrological decision support system model and parameterization for the Lower Mekong River Basin. For example, we were able to report new parameter values needed to estimate evaporation in the SWAT modeling environment (e.g., a soil evaporation compensation factor parameter or ESCO).

We think that our work can serve to improve weather, climate, and hydrological modeling and prediction in the Mekong region. We here call for further research efforts in employing remotely-sensed, satellite-based products for hydrological modeling experiments. Future research should emphasize providing proper guidance for climate forcing data corrections. In addition, investigations into high-resolution soil information data products as well as land use and land cover data are needed.

## Supplementary Material

Supplementary Materials

## Figures and Tables

**Table 1:** The Lower Mekong River annual discharge statistics. Discharge units are in m^3^/s. Country codes are: CN (China), TH (Thailand), LA (Lao People’s Democratic Republic), and KH (Cambodia). The A column refers to the drainage area in square km. *Q_min_* and *Q_max_* refer to the minimum and the maximum annual discharge over the record at each site. The *Q*_1_, *Q*_2_, and *Q*_3_ refer to the 25th, 50th (median), and 75th percentile of the mean annual discharge at each site. The *μ* refers to the mean annual discharge over the record, *σ* is the unbiased standard deviation, *CV* is the coefficient of variation, *σ* is the skewness, *H* is the Hurst coefficient. The coefficient of variation *CV* is equal to *σ/μ*.

Station Name	Code	Country	LMRB	Start Date	End Date	A	*Q_min_*	*Q*_1_	*Q*_2_	*Q*_3_	*Q_max_*	*μ*	*σ*	*CV*	*γ*	*H*
Chinese border	010000	CN	Upper basin inlet	1-Jan-1985	31-Dec-2007	—	1619	2010	2157	2459	2763	2221	303	0.14	0.03	0.35
Chiang Sean	010501	TH	Sub-basin 1 outlet	1-Jan-1960	31-Dec-2016	191,055	1871	2304	2564	2929	4027	2618	427	0.16	0.60	0.72
Luang Prabang	011201	LA	Sub-basin 2 outlet	1-Jan-1939	31-Dec-2016	273,838	1852	3410	3754	4177	5488	3777	707	0.19	−0.12	0.70
Vientiane	011901	LA	Sub-basin 3 outlet	1-Jan-1913	31-Dec-2016	303,528	2677	3975	4455	4900	6111	4476	710	0.16	0.10	0.67
Mukdahan	013402	TH	Sub-basin 4 outlet	1-Jan-1923	31-Dec-2016	394,134	5256	7246	8031	9012	10,496	8071	1168	0.14	−0.02	0.89
Pakse	013901	LA	Sub-basin 5 outlet	1-Jan-1923	31-Dec-2016	550,955	6835	9095	10,050	11,165	12,918	10,066	1434	0.14	−0.08	0.68
Kratie	014901	KH	Sub-basin 6 outlet	1-Jan-1924	31-Dec-2016	656,518	6599	11,891	13,527	15,077	19,562	13,411	2591	0.19	−0.41	0.77
Yasothom	370104	TH	Sub-basin 7 outlet	1-Jan-1952	31-Dec-2003	46,805	77	171	240	287	602	242	102	0.42	1.21	0.60
Rasi Salai	380134	TH	Sub-basin 8 outlet	1-Jan-1979	31-Dec-2003	43,878	5	95	154	223	447	177	107	0.60	0.79	0.94

**Table 2:** Parameters and calibrated values used for the LMRB model simulations. The identifier code refers to replacement (V), addition (A), and multiplication (R).

	Parameter	Description	Range	Identifier Code	Calibrated Value	Calibrated Value

					Remote Sensing Data	In-Situ Data
Precipitation						

	PRECIPITATION	Correction factor to grid precipitation record	−1, +0.01	R	−0.445 to +0.002	−0.983 to −0.007

High Flow						

	CN2	Initial SCS runoff curve number to moisture condition II	−0.1, +0.1	R	−0.07	−0.0315
	AWC	Available water capacity of the soil layer	−0.1, +0.1	R	+0.07	+0.0525
	ESCO	Soil evaporation compensation factor	+0.5, +0.9	V	+0.6	+0.75

Base Flow						

	GWHT	Initial groundwater height	0, +1.0	V	+0.075	+0.425
	GW_DELAY	Groundwater delay time	−30, +60	A	−14.25	−14.25
	GWQMN	Threshold depth of water in the shallow aquifer	−1000, +1000	A	−450	−250
	REVAPMN	Percolation to the deep aquifer to occur	−750, +750	A	+262.5	+337.5
	GW_REVAP	Groundwater “revap” coefficient	+0.02, +0.10	V	+0.042	+0.098
	RCHRG_DP	Deep aquifer percolation fraction	−0.05, +0.05	A	+0.0375	−0.0225

**Table 3:** The LMRB model calibration metric results. The percent error (Qerr) between the monthly mean simulated and observed discharge and the Nash–Sutcliffe Efficiency (NSE) performance metrics are depicted for each sub-basin corresponding to satellite-based remote sensing (RS) and in-situ data input, respectively.

SUB-BASIN	Q_err_ (%)		NSE	

	RS	In-Situ	RS	In-Situ
SB1	0.81	0.53	0.96	0.91
SB2	−0.29	2.02	0.94	0.70
SB3	0.88	−3.31	0.91	0.75
SB4	0.79	−3.41	0.93	0.78
SB5	4.76	5.74	0.94	0.68
SB6	−1.90	−1.64	0.94	0.83

**Table 4:** The LMRB model validation metric results. The LMRB model Nash–Sutcliffe Efficiency (NSE) performance metrics are depicted for each sub-basin corresponding to satellite-based remote sensing (RS) and in-situ data input, respectively.

Sub-Basin	NSE	
	RS	In-Situ
SB1	0.98	0.97
SB2	0.91	0.83
SB3	0.94	0.79
SB4	0.90	0.83
SB5	0.89	0.75
SB6	0.88	0.84

## References

[1] Vörösmarty CJ, McIntyre PB, Gessner MO, Dudgeon D, Prusevich A, Green P, Glidden S, Bunn SE, Sullivan CA, Liermann CR (2010). Global threats to human water security and river biodiversity. Nature.

[2] Ziv G, Baran E, Nam S, Rodríguez-Iturbe I, Levin SA (2012). Trading-off fish biodiversity, food security, and hydropower in the Mekong River Basin. Proc Natl Acad Sci USA.

[3] Zhang B, Zhang L, Guo H, Leinenkugel P, Zhou Y, Li L, Shen Q (2014). Drought impact on vegetation productivity in the Lower Mekong Basin. Int J Remote Sens.

[4] Veilleux JC, Anderson EP (2016). 2015 Snapshot of water security in the Nile, Mekong, and Amazon River Basins. Limnol Oceanogr -Bull.

[5] Winemiller KO, McIntyre PB, Castello L, Fluet-Chouinard E, Giarrizzo T, Nam S, Baird IG, Darwall W, Lujan NK, Harrison I (2016). Balancing hydropower and biodiversity in the Amazon, Congo, and Mekong. Science.

[6] Mekong River Commission (2017). Transboundary Water Resources Management Issues in the Mekong Delta of Cambodia and Vietnam.

[7] Sivapalan M, Blöschl G (2017). The Growth of Hydrological Understanding: Technologies, Ideas, and Societal Needs Shape the Field. Water Resour Res.

[8] Piman T, Lennaerts T, Southalack P (2013). Assessment of hydrological changes in the Lower Mekong Basin from Basin-Wide development scenarios. Hydrol Process.

[9] Piman T, Cochrane TA, Arias ME (2016). Effect of Proposed Large Dams on Water Flows and Hydropower Production in the Sekong, Sesan and Srepok Rivers of the Mekong Basin. River Res Appl.

[10] Lyon SW, King K, Polpanich OU, Lacombe G (2017). Assessing hydrologic changes across the Lower Mekong Basin. J Hydrol Reg Stud.

[11] Li D, Long D, Zhao J, Lu H, Hong Y (2017). Observed changes in flow regimes in the Mekong River Basin. J Hydrol.

[12] Poff NL, Olden JD (2017). Can dams be designed for sustainability?. Science.

[13] Sabo JL, Ruhi A, Holtgrieve GW, Elliott V, Arias ME, Ngor PB, Räsänen TA, Nam S (2017). Designing river flows to improve food security futures in the Lower Mekong Basin. Science.

[14] Rossi CG, Srinivasan R, Jirayoot K, Duc TL, Souvannabouth P, Binh N, Gassman PW (2009). Hydrologic evaluation of the Lower Mekong River Basin with the soil and water assessment tool model. Int Agric Eng J.

[15] FAO; IIASA; ISRIC; ISSCAS; JRC (2012). Harmonized World Soil Database, HWSD (Version 1.21).

[16] Spruce J, Bolten JD, Srinivasan R (2017). Developing land use land cover maps for the Lower Mekong Basin to aid SWAT hydrologic modeling.

[17] WLE (2017). Dataset on the Dams of the Irrawaddy, Mekong, Red and Salween River Basins.

[18] Rodell M, Houser PR, Jambor U, Gottschalck J, Mitchell K, Meng CJ, Arsenault K, Cosgrove B, Radakovich J, Bosilovich M (2004). The global land data assimilation system. Bull Am Meteorol Soc.

[19] Arnold JG, Srinivasan R, Muttiah RS, Williams JR (1998). Large area hydrologic modeling and assessment part I: Model development. J Am Water Resour Assoc.

[20] Srinivasan R, Ramanarayanan TS, Arnold JG, Bednarz ST (1998). Large area hydrologic modeling and assessment part II: Model application. J Am Water Resour Assoc.

[21] Arnold JG, Fohrer N (2005). SWAT2000: Current capabilities and research opportunities in applied watershed modelling. Hydrol Process.

[22] Gassman PW, Reyes MR, Green CH, Arnold JG (2007). The soil and water assessment tool: Historical development, applications, and future research directions. Trans ASABE.

[23] Douglas-Mankin KR, Srinivasan R, Arnold JG (2010). Soil and Water Assessment Tool (SWAT) model: Current developments and applications. Trans ASABE.

[24] Arnold JG, Moriasi DN, Gassman PW, Abbaspour KC, White MJ, Srinivasan R, Santhi C, Harmel RD, Griensven AV, Liew MWV (2012). SWAT: Model use, calibration, and validation. Trans ASABE.

[25] Daggupati P, Yen H, White MJ, Srinivasan R, Arnold JG, Keitzer CS, Sowa SP (2015). Impact of model development, calibration and validation decisions on hydrological simulations in West Lake Erie Basin. Hydrol Process.

[26] Srinivasan R, Arnold JG, Jones CA (1998). Hydrologic modelling of the United States with the soil and water assessment tool. Int J Water Resour Dev.

[27] Abbaspour KC, Rouholahnejad E, Vaghefi S, Srinivasan R, Yang H, Kløve B (2015). A continental-scale hydrology and water quality model for Europe: Calibration and uncertainty of a high-resolution large-scale SWAT model. J Hydrol.

[28] Arnold JG, Kiniry JR, Srinivasan R, Williams JR, Haney EB, Neitsch SL (2013). SWAT 2012 Input/Output Documentation.

[29] Abbaspour KC, Yang J, Maximov I, Siber R, Bogner K, Mieleitner J, Zobrist J, Srinivasan R (2007). Modelling hydrology and water quality in the pre-alpine/alpine Thur watershed using SWAT. J Hydrol.

[30] Hurst HE (1951). Long-term storage capacity of reservoirs. Trans Am Soc Civ Eng.

[31] Weron R (2002). Estimating long-range dependence: Finite sample properties and confidence intervals. Physica A.

[32] Lacombe G, Douangsavanh S, Vogel RM, McCartney M, Chemin Y, Rebelo LM, Sotoukee T (2014). Multivariate power-law models for streamflow prediction in the Mekong Basin. J Hydrol Reg Stud.

[33] Neitsch SL, Arnold JG, Kiniry JR, Williams JR, King KW (2002). Soil and Water Assessment Tool Theoretical Documentation Version 2000.

[34] R Development Core Team (2018). R: A Language and Environment for Statistical Computing, 3.4.4.

