# Supplementary material for: Improved Hydrological Decision Support System for the Lower Mekong River Basin Using Satellite-Based Earth Observations
Source: Remote Sens (Basel). Author manuscript; Available in PMC 2018 Jun 20. (PMC6008643; doi:10.3390/rs10060885)
Supplement: Supplementary Materials [file NIHMS974644-supplement-Supplementary_Materials.zip › Supplementary/Paper_Highlights.docx.zip!/Paper_Highlights.docx]

Highlights

- Satellite data benefit hydrologic modeling at poor spatial in-situ data regions.
- TRMM and GPM have good quality over the southern part of the Lower Mekong River.
- A tool to streamline accessing & processing NASA earth observations data products.
